# BNS: A Framework for Wireless Body Area Network Realistic Simulations

**DOI:** 10.3390/s21165504

**Published:** 2021-08-16

**Authors:** Egberto Caballero, Vinicius Ferreira, Robson Araújo Lima, Julio César Huarachi Soto, Débora Muchaluat-Saade, Célio Albuquerque

**Affiliations:** MídiaCom Lab, Institute of Computing, Fluminense Federal University, Niterói 24210-346, Brazil; vinicius@midiacom.uff.br (V.F.); robsonal@midiacom.uff.br (R.A.L.); jsoto@id.uff.br (J.C.H.S.); debora@midiacom.uff.br (D.M.-S.); celio@midiacom.uff.br (C.A.)

**Keywords:** WBAN, personal health devices, ISO/IEEE 11073, IEEE 802.15.6, e-health

## Abstract

Simulation is a useful and common technique to evaluate the performance of networks when the implementation of a real scenario is not available. Specifically for Wireless Body Area Networks (WBAN), it is crucial to perform evaluations in environments as close as possible to the real conditions of use. To achieve that, simulations must include different protocol layers involved in WBAN and models close to reality to create realistic simulation environments for e-health applications. To satisfy these needs, this work presents the BNS framework, a flexible tool for WBAN simulations. The proposal is an extension of the Castalia framework, which includes: (1) a new wireless channel model considering real radio-propagation over the human body; (2) an updated implementation of the WBAN MAC protocol in Castalia, with functionalities and requirements in accordance with the IEEE 802.15.6 standard; (3) a new comprehensive and configurable mobility model for simulating intra-WBAN communication; (4) a temperature module based on the Pennes bioheat transfer equation, to model the temperature of a WBAN node based on the activity of the node; and (5) a Healthcare Application Layer that implements data representation and a communication protocol between Personal Health Devices (PHD) following the ISO/IEEE 11073 standard. Three use cases are presented, where WBAN scenarios are simulated and evaluated using the proposed BNS framework. Results show that BNS is a valid and flexible tool to evaluate WBAN solutions through simulation.

## 1. Introduction

The miniaturization of devices that can be used as wearables or attached to the human body has made possible the emergence of new communication networks. Thus, Wireless Body Area Networks (WBAN) are networks made up of small devices with low energy consumption and wireless communication capabilities. The main function of this type of network is to monitor and act on human bodies and the environment [1]. The composition of a WBAN network is made by sensor and actuator nodes that interact with a coordinator node. The coordinator node is responsible for collecting and sending the required information to the sensor/actuator nodes, in turn, the coordinator node offers communication with other types of networks. The nodes of a WBAN can collect data of vital and physiological signals, monitoring, processing, and communicating those data in real time without causing discomfort to patients.

The increase in older adults and patients with congenital, degenerative and other diseases, together with the medical expenses that they cause, resulted in the appearance of new technologies. Thus, WBAN was created to be applied to healthcare (e-health) for monitoring patients at a low cost and thus alleviating medical and economical expenses. However, the use of WBAN is not restricted to the health area and can be applied to other fields such as entertainment, sports, military services and others [2].

IEEE 802.15.6 [3] published in 2012 is the standard that best defines WBAN, which specifies wireless communications on and around the human body. The standard defines an architecture with three levels: Intra-WBAN, Inter-WBAN and Beyond-WBAN. IEEE 802.15.6 defines the functionalities of the Physical (PHY) and Medium Access Control (MAC) layers. In addition, it shows its main characteristics related to low energy consumption, reliability and transmission rate. We also find definitions of network topology, such as star topology, extended star and security levels with different security and protection properties [4]. After the IEEE 802.15.6 standard publication, research work focused on layers not defined in the standard. However, more recent studies propose modifications to the WBAN standard. For example, in [5], modifications to the MAC layer is proposed. The results of that work show that the proposed protocol works better than the WBAN standard in terms of throughput, energy consumption and packet delivery delay.

Together with the advances of WBANs, new social and technical challenges arise. Ease of use, data security and privacy, interoperability, cost, physical security, and user well-being are social challenges to be overcome. Technically the wireless channel dynamics, the computational power limitations, the energy efficiency and the operation of the transceiver are the main challenges. Other challenges such as device overheating, heterogeneous environment, Quality of Service (QoS) provision must be evaluated in WBAN to allow practical adoption [6,7].

Another interesting point of WBANs is the heterogeneity of their components, i.e., for monitoring vital and physiological signals, different types of sensor nodes are needed that clearly differ in computational resources, storage capacity, energy consumption and sampling rates. In addition, wireless communication between sensor nodes is carried out through the human body, which leads to its degradation, causing in many cases the fading of the communication channel, loss of trajectory, and high latency. Additionally, the movement of the human body, such as the movement of arms and legs can cause the breakdown of the network topology. Thus, we say that the environment where WBAN communication takes place is highly dynamic and unstable, where a specific communication channel must be considered that guarantees the good performance of communication in highly dynamic and unstable environments [4].

The increase in temperature is one of the most relevant challenges for WBAN. For implanted nodes, a dramatic increase in temperature heats the tissue around the node and can cause tissue damage and infection. For example, in [8], it is exposed that a temperature increase of 0.1 °C is sufficient to activate thermoregulatory responses of the human body that can be harmful in the long-term. In WBAN scenarios, two sources of a node’s temperature rise are: the transceiver radiation and energy consumption of the node’s circuits. Using low transmission power, the radiation absorption is reduced, and avoiding long periods of node operation, the increase in node temperature may be controlled [9].

Specific protocols for WBAN have been proposed to overcome these technical challenges. Whenever a new technological proposal emerges, it is important to carry out performance evaluations in environments in which they will be used. However, it is not always feasible to create the infrastructure for a real test scenario; whether for economic reasons or other human factors. In such cases, evaluating a new solution using simulation is the preferred option. Therefore, it is necessary to have models as close to reality as possible to create realistic simulation environments to evaluate the performance of new WBAN proposals.

In the literature, several simulation tools are used to evaluate WBAN proposals, such as OMNET++, Castalia [10,11,12], MiXiM [13], NS-2 [14,15], NS-3 [16] and MatLab [17,18,19,20,21]. In addition to simulation tools, there are test-bench-based studies such as [22,23]. Most simulation tools focus on a single network layer evaluation. For example, most MatLab simulations aim at modeling the PHY and MAC layers. However, studies like [20] also use MatLab to evaluate the performance of routing proposals. On the other hand, network simulators such as NS-2 and OMNET++ are often used to evaluate proposals for routing protocols. This dissociation between layers reduces the realistic likelihood of the simulated scenario.

Therefore, there is a need for a simulation tool that integrates all network layers and at the same time satisfies the demands of WBAN. Specifically, Castalia [10] is a framework that addresses this need for integration of layers. Castalia became the most widely accepted proposal in studies focusing on WBAN, mainly because it offers an experimental channel model and an implementation of the IEEE 802.15.6 *draft* version. However, to simulate WBAN in realistic environments, Castalia still needs to incorporate new modules, such as the effect of node transmissions on body temperature, human body mobility models, specific routing protocols, and a healthcare standard for the application layer. The WBAN standard defines an architecture with three levels: Intra-WBAN, Inter-WBAN and Beyond-WBAN [3]. In the application layer, the current version of Castalia only considers configurations for generic Wireless Sensor Network (WSN) devices without considering the particularities of healthcare sensors. Additionally, the data representation and the communication mechanisms between the sensor nodes and the coordinator node for e-health applications, specified in the IEEE-11073 standard, are not considered.

To fill in those gaps, this work presents the Body Network Simulator (BNS) framework, an extension of the Castalia framework for intra-WBAN communication, which includes:

- Wireless Channel Model:The wireless channel model Goswami et al. [24], created specifically for WBAN is implemented and available in the BNS framework.
- IEEE 802.15.6: The draft version of the MAC protocol provided by Castalia is updated with functionalities and requirements of the IEEE 802.15.6 standard published in 2012.
- Mobility Model: A mobility model based on MoBAN [25] is also provided in the BNS framework. The model enables the use of a set of postures and node’s movements based on the current posture and node placement.
- Temperature Model: A temperature model based on the Pennes bioheat transfer equation [26] is incorporated. This model calculates the temperature variation based on the activity of the node. Moreover, the SAR generated by the transceiver radiation and the power dissipated by the nodes’ electronic components are considered heat sources.
- Healthcare Application Layer: This proposal implements the data representation and the communication protocol among the application layers of Personal Health Device (PHD) according to the ISO/IEEE 11073 standard  [27]. This application layer provides five different types of PHD to act as real ISO/IEEE 11073 devices (X73-PHD) in WBAN simulations: pulse oximeter, glucose meter, thermometer, blood pressure monitor and a basic Electrocardiogram (ECG) sensor. The Antidote Library [28] was used to assist in the development of this application layer. The Antidote Library was developed to be used in real medical devices as part of the SigHealth Platform [28], a platform for remote patient monitoring and data management using personal wireless devices for health. A plug-in that allows communication between the Antidote Library and the BNS framework was developed.

The remainder of this paper is organized as follows. Section 2 presents a review of tools for WBAN simulation used in the literature. Section 3 presents the proposed BNS framework and the implementation of the contributions presented in this work. Section 4 discusses the results of some use cases of the proposed BNS framework in WBAN research and Section 5 summarizes the conclusions and future work.

## 2. Related Work

Several simulations tools are used to evaluate WBAN proposals, such as OMNET++, Castalia, MiXiM, NS-2, NS-3 and MatLab. In addition to simulation tools, there are test-bench-based studies [22,23], and others where authors developed their own simulators [29].

OMNET++ [30] is a discrete-event simulator commonly used to build network simulators. It has an object-oriented modular structure and is developed in C++. Its module-based architecture allows it to be easily used in new network simulator proposals. Among the frameworks for network simulation developed on top of OMNET++ are MiXiM [13] and Castalia [10]. MiXiM is an OMNET++ modeling framework created for mobile and fixed wireless networks as WSN, WBAN, and others. It offers detailed models of radio wave propagation, interference estimation, radio transceiver power consumption and wireless MAC protocols. However, the implementation of the WBAN standard is not available.

Castalia framework [31] is more specific for simulating WBAN protocols. It was developed for low-power device networks. Specifically, to simulate WBAN, Castalia offers an advanced channel model based on empirically measured data for the human body as the propagation medium as well as a radio model based on real radios for low-power communication. Additionally, the radio model is highly flexible. It estimates the reception probability based on Signal-to-Interference-plus-Noise Ratio (SINR), packet size and modulation type. It allows several levels of transmission power, being able to identify variations for individual nodes. Castalia performs a realistic modeling of the Received Signal Strength Indicator (RSSI) and carrier detection, it defines states with different energy consumption and delays in switching between states and considers the node’s clock deviation [10]. Moreover, Castalia has an implementation of the IEEE 802.15.6 *draft* version. Because of that, Castalia became the most widely accepted proposal in studies focusing on WBAN [12,32,33,34,35,36,37].

For example, in [37] Castalia is used to evaluate the performance of the PHY, MAC and Network layers of WBAN. In [36] a comparative analysis of the IEEE 802.15.4 [38] and IEEE 802.15.6 standards over a WBAN healthcare monitoring system is performed using Castalia, to evaluate QoS parameters of the two standards under the same conditions. However, Castalia does not provide necessary modules for realistic simulations, such as: body temperature model, WBAN specific routing protocols, and a Healthcare Application Layer.

NS-2 is an open source, discrete-event network simulator. It was built in C++ and is capable of simulating wired as well as wireless networks. Some researchers, focusing on routing algorithms for WBAN, have used NS-2 to simulate and evaluate their proposals. In [15] a stable, reliable and energy efficient routing protocol for WBAN is proposed. In that work, the authors evaluated two performance metrics, energy consumption and routing overhead. Their proposal was implemented and evaluated using NS-2. In [14] the authors propose DMQoS protocol. In this work, the performance of the DMQoS is compared with DARA [39] and LOCALMOR [40] protocols, other routing protocols developed in NS-2. In those studies, simulations were focused on evaluating the performance of the proposed protocol and the IEEE 802.15.4 standard was used in the MAC layer. In the literature, no work has been identified with the implementation in NS-2 of the IEEE 802.15.6 standard MAC layer.

The NS-3 project [41] was initiated in 2006. Like NS-2, is an open source, discrete-event network simulator. This simulator is considered to be a replacement of NS-2 and not an extension. It is written in C++ language and python. Some studies have implemented models to simulate realistic WBAN scenarios using NS-3. For example, in [42,43], implementations of the IEEE 802.15.6 standard are proposed for NS-3. Additionally, in [16] a comprehensive review of the main components of the IEEE 802.15.6 standard is conducted and modeling strategies are presented to implement IEEE 802.15.6 MAC in the NS-3 simulator. In that work, the authors also modeled the necessary components in NS-3 to provide a realistic simulation environment and to reliably evaluate health monitoring systems. Among these components, we find models for PHY, MAC, error rate, propagation loss, on-body channel, energy, and human mobility. However, a temperature model that allows monitoring the heating of the WBAN sensor nodes and an application layer that implements the communication protocols between personal health devices in correspondence with the ISO/IEEE 11073 standard are not provided for NS-3. Additionally, there is no freely available WBAN mobility model for simulating WBAN in any of those simulation tools [25].

MatLab is a powerful simulation tool, and is also used in several studies focused on WBAN [17,18,19,20,21]. However, it does not allow simulations to be performed considering all network layers. Matlab is most used in studies focused on modeling the PHY and MAC layers. However, proposals like [17,20] also use MatLab to evaluate the performance of routing protocols. In [17] a thermal-aware routing protocol that avoids high temperature areas (hot spots) is proposed. In their work, a temperature model is implemented; however, in the study only the performance of the proposed routing protocol is evaluated without considering other layers. It is important to note that MatLab is not a network simulator itself. It allows implementation, simulation and evaluation of the behavior of models. However, for network simulation there are better tools such as OMNET++.

In other studies, prototypes of WBAN nodes are used to evaluate communication protocols and the wireless channel. For example, in [22] they explore on-body packet routing issues in the presence of topological partitioning caused due to ultra-short wireless range and postural body movements. In that work, a probabilistic packet routing protocol is then developed using a stochastic link cost, reflecting the body postural trends. The performance of the proposed protocol is evaluated experimentally. For that, authors developed a prototype of WBAN. In [23] the behavior of the wireless channel is estimated in correspondence with a real scenario. As a main result of that work, a loss map and a temporal variation model of the wireless channel are proposed, which were generated based on real measurements. For that, experiments were carried out using transmitting and receiving antennas on the body of people, simulating the transceivers of the WBAN nodes. In those work, WBAN prototypes do not consider a protocol architecture with all layers of a WBAN network. However, implementing a WBAN for testing can prove costly and in addition, testing real-world scenarios that consider nodes implanted in the body remains a challenge.

In [29], a thermal-aware routing protocol is proposed. The objective of that work is to reduce overheating of sensors implanted in human bodies. The authors evaluated the temperature increase and the energetic efficiency. For that, the authors developed a specific simulation program in Java using discrete-event simulation. However, the simulator does not comply with the definitions of the WBAN standard.

In the literature review, the need for simulation tools that provide an application layer with communication protocols for real personal health devices was identified. In addition, the different simulation tools have some dissociation between the different layers of the proposed network architecture for WBAN, which reduces the likelihood of simulating a real scenario and makes comparison difficult. To overcome these issues, this work presents the BNS framework as an extension of Castalia. Details of the implementation modules are presented in Section 3. Table 1 shows a comparative summary of the simulation capacity of WBAN networks for the different simulation tools referenced in the literature and the BNS framework proposed in this work.

## 3. Proposed BNS Framework

**Figure 1:**
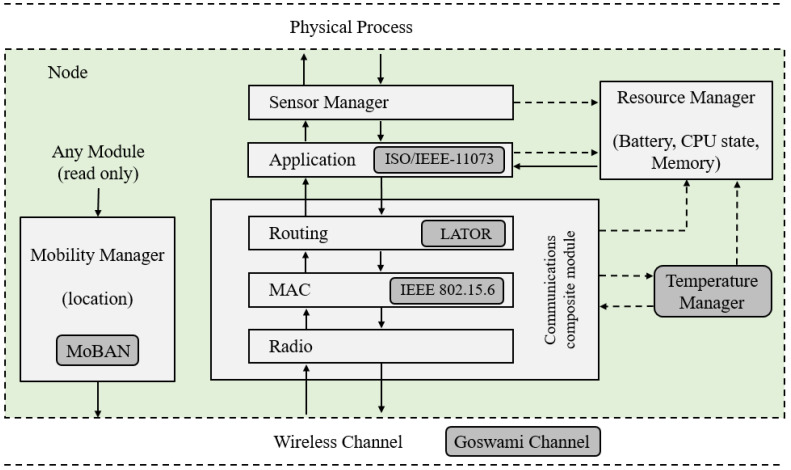
The BNS framework structure.

Our proposed BNS framework for WBAN simulations (Available online: https://github.com/midiacom/BNS-Framework (accessed on 10 August 2021)) has a hierarchical structure based on modules. The BNS framework structure is shown in Figure 1. There are three main interconnected modules: the node, the wireless channel and the physical process. In the BNS framework, the nodes are not directly connected to each other, but through the wireless channel module. When a node has a packet to send, it goes to the wireless channel, which decides which nodes should receive the packet. Additionally, nodes are linked to the physical process, from which they obtain information about some physical magnitude collected by the sensor.

The BNS framework represents each WBAN node as a composite module, which is formed by several sub-modules that represent the different layers of the WBAN network, and others auxiliary sub-modules that model important features of WBAN. As it can be seen in Figure 1, each node module contains: Temperature Manager, Resource Manager, Mobility Manager, Sensor Manager, Application and Communication sub-modules. In addition, Communication sub-module contains Routing, MAC and Radio sub-modules. In this figure, the solid arrows represent the exchange of messages between the different sub-modules and the dashed arrows represent simple function calls. For example, most modules call a resource manager function to indicate that the energy has been consumed.

The BNS framework is an extension of Castalia and, in this proposal, new available modules are represented in Figure 1 with grey boxes. A model of channel behavior for WBAN proposed by Goswami et al. [24] is implemented. The Mobility Model for Wireless Body Area Networks (MoBAN) proposed by Nabi et al. [25] is implemented as a new Mobility Manager sub-module. A new temperature module is implemented. This allows estimation of the temperature of the nodes based on their activity. The Temperature Manager module makes function calls to the resource manager and radio modules to have the activity information of the node and to be able to calculate the temperature of the node. In addition, sub-modules of the Communication module can consult the node temperature.

The MAC layer sub-module corresponding to the draft WBAN standard is updated with features and requirements of the IEEE 802.15.6 standard published in 2012. In the routing sub-module, the Link-Quality Aware and Thermal Aware On-Demand Routing (LATOR) protocol proposed in [9] is added. This protocol uses the temperature calculated by the Temperature Manager module to avoid overheating the nodes. In the Application module, a Healthcare Application sub-module is added. This one implements data representation and a communication protocol of PHD recommended in the ISO/IEEE 11073 standard. Details of these implementations will be presented in the following sections. The BNS framework uses an infrastructure of abstract classes and interfaces. This infrastructure is extensible, new modules, sub-modules and functionalities can be implemented and added to the existing framework.

### 3.1. Wireless Channel

Ultra-wideband Physical Layer (UWB-PHY) allows low-power transmission, low-power consumption, high data rate, and robustness against multi-path fading. That makes it a suitable air interface for WBAN systems. The IEEE 802.15.6 standard includes UWB-PHY. There are many attempts to model the WBAN wireless channel [24,44,45,46]. In [24], Gowami et al. experimentally determined a model for the path loss of on-body Ultra-wideband (UWB) channel. That model was statistically determined from measurement data and has parameters for link obstruction (Line-of-Sight (LOS) and Non-Line-of-Sight (NLOS)), different body shapes (underweight, normal, and overweight), and each UWB high band channel of the IEEE 802.15.6 standard. Therefore, we chose to implement the Gowami et al. channel modeling [24] to provide flexibility and different simulations scenarios.

The Goswami channel model [24] provides a description of the channel behavior around the human body, since they used a considered number of receiving antennas spread over the body with a separation of 5 cm between two consecutive nodes. This model considers the LOS and NLOS channel conditions over the human body. It also considers the influence of people’s physiques on the loss of path. For this, people with different weights and classified as ‘thin’, ‘normal’ and ‘fat’ participated in the experiment. With MatLab software, the frequency response and the average path loss for each sub-band defined in the WBAN standard were obtained. The path loss is modeled as a log-normal distribution, and the distance dependent path loss is expressed as in Equation (1). The reference distance ($d_{0}$) is 5 cm, the path loss for that reference distance ($Pl(d_{0})$), the path loss exponent (*n*), and the shadow fading (χ) are given for different scenarios.

(1) $$Pl_{dB}\left(d\right)=Pl_{dB}\left(d_{0}\right)+10\cdot n\cdot log_{10}\left(\frac{d}{d_{0}}\right)+\chi$$

**Figure 2:**
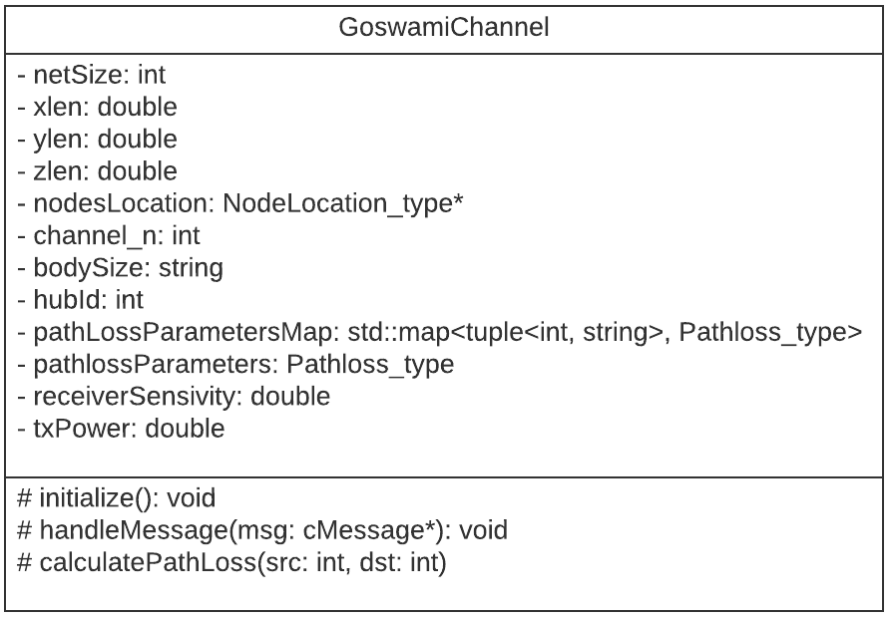
Goswami channel class implemented in the BNS framework. The character “*” refers to pointer-type variable.

As part of our work, the Goswami channel model is implemented and now provided in the BNS framework. Figure 2 shows the Goswami channel class implemented. The attributes are the size of the network, the dimensions of the simulated area, a list of node positions, the channel used, the body size, a map of channel/body size to path loss parameters, a structure of path loss parameters for LOS and NLOS scenarios, the radio sensitivity, the transmission power, and the hub identifier. The *Pathloss_type* structure stores parameters of the log-normal channel, which contains the loss exponent, the reception power at a given reference distance, the reference distance, and the standard deviation value to generate the random variable, for both LOS and NLOS channels.

There are three main methods: the initialization method, which is responsible for reading the parameters from the configuration file and initializing the variables; the handleMessage method that receives node movement messages from the mobility module, and messages of start and end of a wireless transmission; and the method that calculates the path loss between two nodes. In the implemented model, the hub is a position reference on the Cartesian axis and each node in front of it in the direction of the walking movement (+z) is considered to be in its LOS. When the node is behind the hub, the body is considered to be an obstruction and the channel model with NLOS is used for the path loss calculation.

Previous studies only consider the Narrow Band Physical Layer (NB-PHY), using a David channel model [23] available in Castalia. David channel model is an advanced model based on empirically obtained data. This model defines a map of path losses, instead of simple connections between nodes. It consists of a model for temporary variation of path loss and supports node mobility. This model is the result of the work presented in [23], where experiments were carried out using transmitting and receiving antennas on the bodies of eight adults, simulating the transceiver of a WBAN node. Six transceivers were considered and allocated in the following positions: one on the right side of the waist, one on each wrist, one on each foot and one on the chest.

This study just consider a NB-PHY. Our BNS framework, also provides a David channel model. Therefore, the user can choose the desired physical layer for the scenario to simulate, NB-PHY or UWB-PHY. Another important aspect is that David channel model is limited to the number of nodes and the positions used in the experiment. On the other hand, the Goswami wireless channel considers a larger number of nodes allocated on the body. Since the BNS framework provides both models, BNS offers more flexibility to simulate WBAN scenarios.

### 3.2. Mac Layer

The BNS framework MAC Layer implementation extends the draft version available in Castalia to meet the requirements of the frame format of the IEEE 802.15.6 standard published in 2012. In the BNS framework MAC Layer, we added features such as: storing the list of neighboring nodes and some metrics of communication between neighbors; the possibility of encapsulating frames and using nodes as relay; prioritization of traffic when using Carrier Sense Multiple Access with Collision Avoidance (CSMA/CA) medium access method; and the adjustment of allocation times, channel assessment and other temporal parameters to follow those recommended by IEEE 802.15.6 NB-PHY. The changes in the frame header fields were as follows:

- Changing the structure of source and destination fields. The draft version of the standard did not provide the possibility of communication between network nodes, and was restricted to the node/hub communication. Therefore, in the implementation provided in the simulator there were only two markers, one for the address of the hub (*Hub ID*) and one for the address of the *Node ID* node, which were used to identify the hub and node in the communication indiscriminately whether source or destination. To enable the communication between different nodes, those two fields were replaced by the three used in the standard header: *Recipient ID*, *Sender ID* and *BAN ID*. In this way, it is possible to identify the message source and destination in a BAN, and permits node/node communication.
- Inclusion of the *BAN Security/Relay* field. The *BAN Security/Relay* field is a flag that, if used by the hub, defines whether the BAN accepts only secure connections or also accepts insecure ones, features that are not implemented in the simulator. However, when used by network nodes, the field is interpreted as a relay flag, marking an extended topology frame. Within the scope of this work, other implementations were made that allow the use of the field as relay flag.
- Inclusion of other fields. For the header to have the size and all fields specified in the standard, the fields *Last Frame/Access Mode/B2* and *Non-final Fragment/Cancel/Scale/ Inactive* were also inserted, but without the implementation of its functionalities.

**Figure 3:**
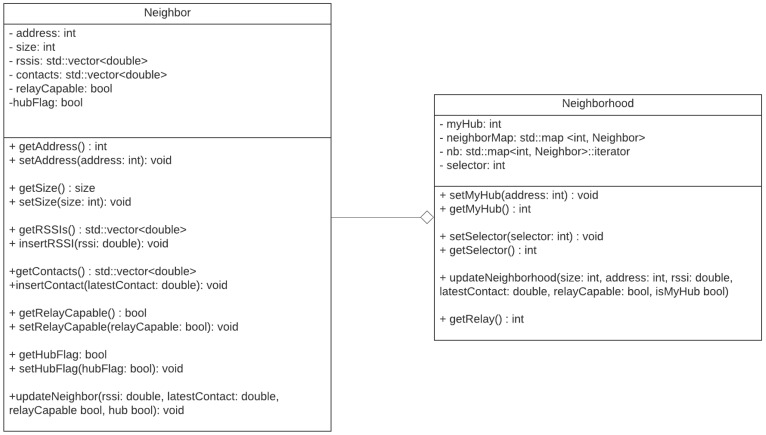
Class diagram for the IEEE 802.15.6 MAC module implemented in the BNS framework.

To enable the use of the relay functionality, in addition to the *BAN Security/Relay* field, the framework subtypes *Relay Request*, *Relay Accept* and *Relay Refuse* were also implemented, and methods for encapsulating and decapsulating frames. Thus, it is possible to investigate the use of the extended topology in future work. To enable the monitoring of neighboring nodes, a class structure was created to store their information. The created classes are represented in the diagram of Figure 3.

With this new class structure, it is possible to store information about neighboring nodes, such as their address, whether they can be used as relay, if the node is a hub and in addition to two vectors of *size* store the instant of the last contacts and the RSSI of the received frames. In addition to this node information, a neighborhood structure makes it possible to verify this stored information through a map that uses the address as a key and for the functions of relay an attribute called *selector*, which makes it possible to use different criteria for selecting a relay node, which can be done by a neighbor with a better RSSI or with the most recent contact. In addition to those modifications, the random-access priority level was implemented and available in the BNS framework. The IEEE 802.1p classes of services are used, and each class has a contention window interval, defined by the IEEE 802.15.6 standard. Other small corrections were also made, such as:

- The addressing scheme: A range of addresses exclusive to nodes connected is used, making it possible to distinguish whether the frames are from nodes connected to the network or not, and the possibility of using 256 connected nodes, as indicated by the standard.
- The use of carrier sensing intervals and allocation slot lengths defined by the IEEE 802.15.6 NB-PHY standard.

### 3.3. Mobility Model

In WBAN, network topology can often vary due to the human body mobility. Furthermore, the quality of the channel between nodes varies according to the relative position of the sensor nodes. That is why accurate body mobility models are important for WBAN. The BNS framework provides a comprehensive and configurable mobility model for individual mobility, simulating intra-WBAN communication. This mobility model is MoBAN [25]. It determines the node positions at any instance of simulation time and so influences the network topology and link properties. MoBAN is built by two basic control units, which are the posture selector and movement module. The posture selector process determines the current posture at any instance of time; and then, according to the selected posture the individual movement of the node is determined.

In [25], authors used an abstraction based on a Markov model to decide about the posture at any time considering the current posture and a transition probability matrix. Then, when a mobile posture is selected, a target position is chosen to start the movement of the whole WBAN. Additionally, spatial correlation was taken into account in MoBAN through the possibility of specifying location-dependent distributions for the posture pattern (Markov chain), and WBAN target position selection. Our implementation in the BNS framework follows the same principles.

After selecting the posture, each node performs movements relative to its position in the body, in addition to individual random movements in the implementation of the proposed mobility model, focused on the individual movements for different postures. To this end, a marker was created for the position of the nodes in the body, with the following markings: head, left arm, left hand, chest, right arm, right hand, center of the waist, right of the waist, left leg, left foot, right leg, right foot. The positioning marks of the nodes follow the positions proposed in the channel model of Van Roy et al. [44]. Thus, there is the possibility of using this channel model in conjunction with the mobility model. These markings are used as a reference for performing periodic movements for nodes in the legs, arms, hands and feet when the selected posture is walking or running. In other positions and postures, only random movement occurs.

**Figure 4:**
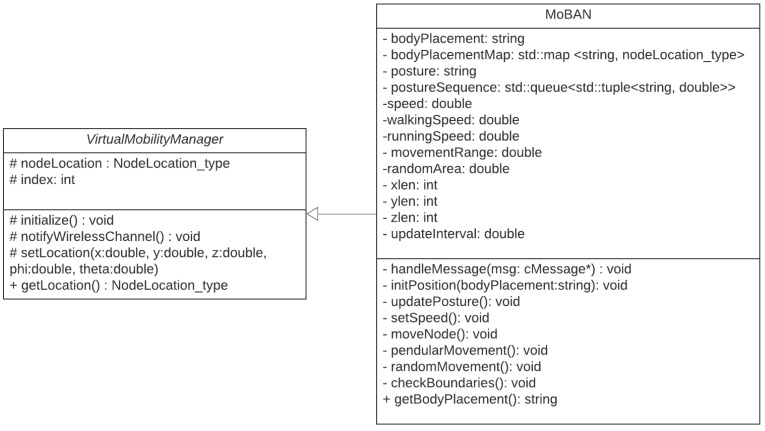
Class diagram of the mobility model implemented in the BNS framework.

The class diagram in Figure 4 describes our implemented classes available in the BNS framework. The *VirtualMobilityManager* is the virtual class with basic features to develop a mobility model. It has a structure for the location of the node, which stores the Cartesian coordinates and allows the use of spherical coordinates. It also has a node identifier and the methods for initializing and reading the parameters of the simulation configuration file, sending messages to the wireless channel module to notify changes in node position.

For the implementation of MoBAN in the BNS framework, a body position identifier *bodyPlacement* was added and a map was created to associate this position *string* with a node location, so when the model is initialized, the *initPosition* method places the node. There is also a queue of postures and the respective time for the posture to be adopted. We also defined the speed of the node, to differentiate if it is walking or running, the range of motion in the case of pendulum movement, the random movement area, the simulation area thresholds and the interval for updating the position of the node.

Therefore, two timers are used, one scheduled at each *updateInterval* to update the node position, which when triggered calls the method *moveNode()*, notifies the wireless channel, schedules the next update; while the other timer is used for posture update, responsible for changing the posture string, updating the node speed and scheduling a new timer for the next posture change. The *moveNode()* procedure checks the posture and position of the nodes and calls the specific movement functions for it. The implementation in the BNS framework follows Algorithm 1. In this way, each node performs only the movements necessary for its position in the given posture. *PendularMovement* makes pendular movements around the node’s initial location in a range defined by *movementRange()*, *randomMovement()* performs random movement within a radius defined by *randomArea* in around the current location of the node and *checkBoundaries* checks whether, after the movements, the nodes have exceeded the simulation area, limiting the movement to the threshold of the simulation area.
**Algorithm 1** moveNode().1:**if**posture= walking **ou** running **then**2: **if**  bodyPlacement = rightArm  **or**  rightHand  **or**  leftArm  **or**  leftHand  **or**  rightLeg **or**  rightFoot  **or**  leftLeg  **or**  leftFoot  **then**3:  pendularMovement()4: **end if**5:**end if**6:randomMovement()7:checkBoundaries()

### 3.4. Temperature Manager

The BNS framework provides a Temperature Manager Module to model the temperature variation. This module implements the Pennes Bioheat Transfer Equation (BHTE) [17], which describes heat transfer in living tissue. With BHTE, it is possible to estimate the temperature (in ${}^{\circ }$C) every instant of time at any point in the tissue around an implanted node. BHTE is presented in Equation (2): ρ (kg/m^3^) is the tissue density, $C_{p}$ (J/(kg ${}^{\circ }$C) is the specific heat of the tissue, *K* (J/m s ${}^{\circ }$C) is the thermal conductivity of the tissue, *b* (J/m^3^ s ${}^{\circ }$C) is the blood perfusion constant and $T_{b}$ (${}^{\circ }$C) is the blood temperature.

Specific heat is defined as the amount of heat that must be supplied to a unit of mass (1 kg) of a tissue to raise its temperature in 1 ${}^{\circ }$C. It can be measured calorimetrically. However, determining the specific heat for each type of tissue in the human body is not a trivial task. Many studies have been developed for this purpose, making this data available in the literature. In [47] a specific heat of different human and animal tissues is given.

In Equation (2), the term $\frac{\partial T}{\partial t}$ indicates the temperature increase in the control volume, $K{▽}^{2}T$ indicates the heat accumulated by the tissue, $b\left(T-T_{b}\right)$ represents the heat transmitted due to the conduction and perfusion of the blood, $Q_{m}$ (W/m${}^{2}$) the heat generated by metabolism, *Q* the heat generated by external sources. As shown in Equation (3) for WBAN, two sources are considered: ρSAR is the heat generated by the antenna radiation, where SAR (W/kg) is the Specific Absorption Rate; $P_{c}$ (mW) indicates the heat due to the node’s power dissipation. In BNS, we implemented the discrete form of BHTE according to [9].

(2) $$\rho C_{p}\frac{\partial T}{\partial t}=K{▽}^{2}T-b\left(T-T_{b}\right)+Q+Q_{m}$$

(3) $$Q=\rho SAR+P_{c}$$

In the proposed temperature model available in the BNS framework, it is necessary to know, for each time interval ($\delta_{t}$), the energy consumed by the node and the radio transmission time. To ensure data collection and temperature update in the $\delta_{t}$ interval, a timer is used. To obtain the consumed energy, the *getSpentEnergy()* method is used in the resource manager module and the *getTxTimeInLastSlotTime()* method in the radio module is used to obtain the radio transmission time. Both methods were implemented in the corresponding modules.

**Figure 5:**
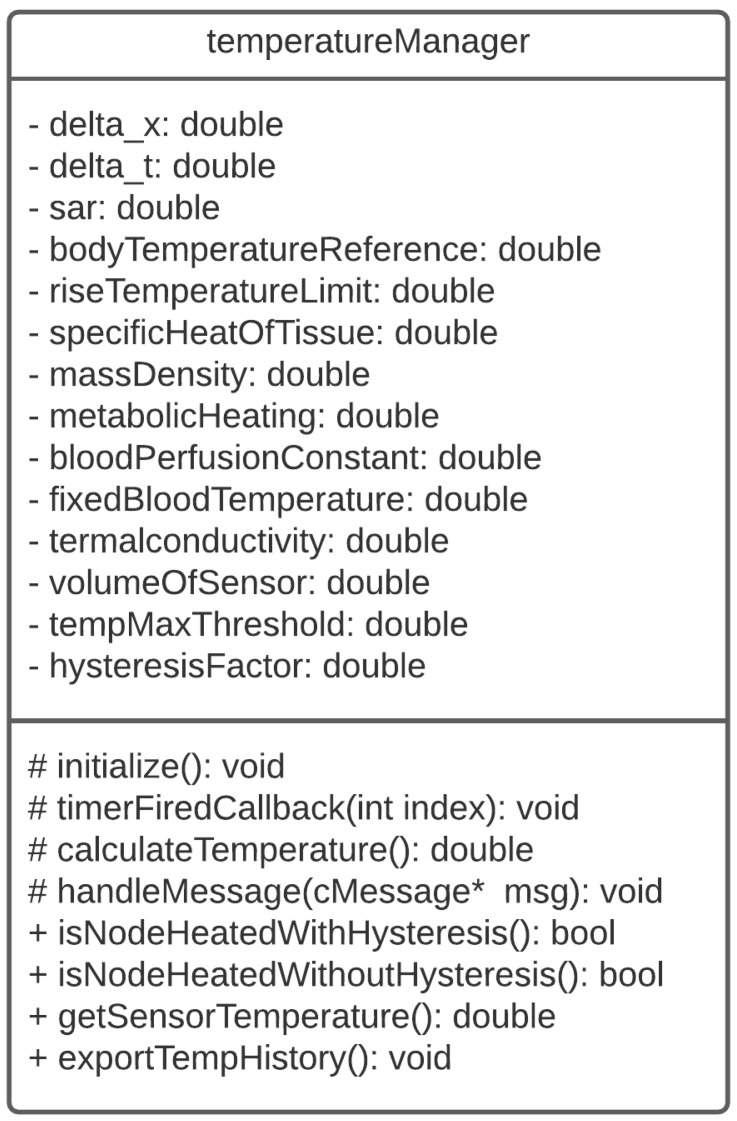
Class diagram of the BNS temperature model. The character “*” refers to pointer-type variables.

As it can be seen in Figure 5, the *TemperatureManager* class implemented in the BNS framework has a set of attributes that correspond to the thermal and radio-electric parameters of the body tissue, in which the node will be implanted. It also provides the *getSensorTemperature()* method, which can be used by the other modules to know the temperature of the node. The *exportTempHistory()* method allows you to export the temperature history of the nodes to be processed after simulation.

The BNS temperature module, in addition to specifically providing the node temperature, also allows one to know the heating state of the node. For this, a maximum allowed temperature value (*tempMaxThreshold*) is defined, from which the node is considered to be HEATED. In the model, a hysteresis interval is also considered for the node to return to the NOT HEATED state again after reaching heating. The hysteresis value of the temperature is defined as a function of *hysteresisFactor* percent of the maximum allowed temperature. The model also allows the possibility of knowing the heating state of the node considering or not the hysteresis interval.

### 3.5. Healthcare Application Layer

In this work a Healthcare Application Layer for the BNS framework is implemented. It implements data representation and a communication protocol between the application layers of PHD in correspondence with the ISO/IEEE 11073 standard [27]. Therefore, real traffic ISO/IEEE 11073 Personal Health Devices (X73-PHD) for WBAN are implemented. This standard defines two types of PHD: agents and managers. The agent devices are sensors or actuators and the manager device corresponds to the network coordinator. Additionally, BNS has different specifications for each PHD, called specializations. Specializations define a set of standards for each of the PHD. Each specialization has its own Domain Information Model (DIM), i.e., which attributes and objects a device must have. It is important to define a DIM separately for each device, since an electrocardiogram has different technical needs than a pulse oximeter. All specializations follow a unique service and communication model, defined in the Optimized Exchange Protocol (OEP). The OEP protocol is the heart of the ISO/IEEE 11073 standard and is where the DIMs common to all devices are defined, as well as the service model and the communication model [1].

The BNS Healthcare Application Layer provides five different X73-PHD agents for WBAN simulations: pulse oximeter, glucose meter, thermometer, blood pressure monitor and a basic ECG. Their implementation in BNS follows the specifications for transmission rate and sample generation as described in [1].

Following the ISO/IEEE 11073 standard, the BNS Healthcare Application Layer is agent and manager-initiated, providing the *confirmed* and *unconfirmed events*, and defining three modes of manager-initiated measurement data transmission: *Single Response*, *Time Period* and *No Time Limit Mode*. All these modes defined in the standard are covered in [1] and implemented in the Healthcare Application Layer provided in the BNS framework.

The BNS framework also provides the *Retransmission Mode* proposed in [1]. This mode is a new proposal, not present in the ISO/IEEE 11073 standard. *Retransmission Mode* consists of a stop-and-wait system as a sub-application-layer to retransmit agent packets whose Acknowledgments (ACKs) have not been received. This interesting solution covered in [1] reduces the unnecessary exchange of several control packets made in association procedures. In addition, it is an alternative to fill the lack of a reliable transport layer in the ISO/IEEE 11073 standard.

In the Healthcare Application Layer proposed in the BNS framework, the user can set several simulation parameters, such as the medical device type, operation mode and the number of transmission retries. Table 2 summarizes all parameters and their possible values are shown.

#### Implementation of the Application Layer

Antidote [28] is an IEEE 11073 protocol stack library developed to work with real medical and health devices. To provide communication between Antidote and other systems, communication plug-ins are required, because it has a plug-in-based architecture. For that, a BNS plug-in was developed to support communication between Antidote and the BNS framework modules. In addition, some necessary modifications to the communication, encoders, agent and manager modules of the Antidote, covered in [1], are implemented in the BNS framework.

The proposed BNS plug-in allows the BNS framework and Antidote to talk with each other. Inside the BNS framework, we can call any function from the Antidote Library and retrieve any information from it, but transmitting data from the BNS framework to the Antidote Library is not a simple task. To solve this, we first use external variables to make the data exchange between the BNS framework and the BNS plug-in module. Then, to obtain the data from the BNS plug-in, Antidote uses pointers to the plug-in functions that we call *callback functions*. That is why a plug-in to mediate the communication is needed.

**Figure 6:**
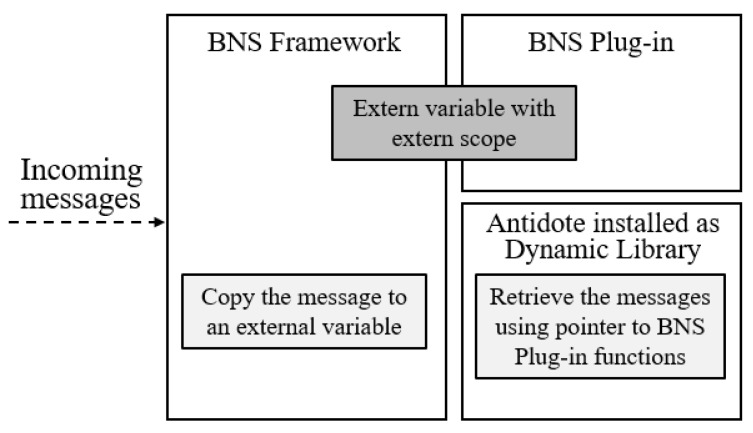
How Antidote Library receives messages from the BNS Framework.

Figure 6 depicts how Antidote receives messages from the BNS framework. The arriving messages are copied to an external variable in the BNS framework. The external variable is visible to both BNS framework and BNS plug-in. Since the Communication module in Antidote owns pointers to the BNS plug-in functions, it can retrieve the messages using callback functions.

**Figure 7:**
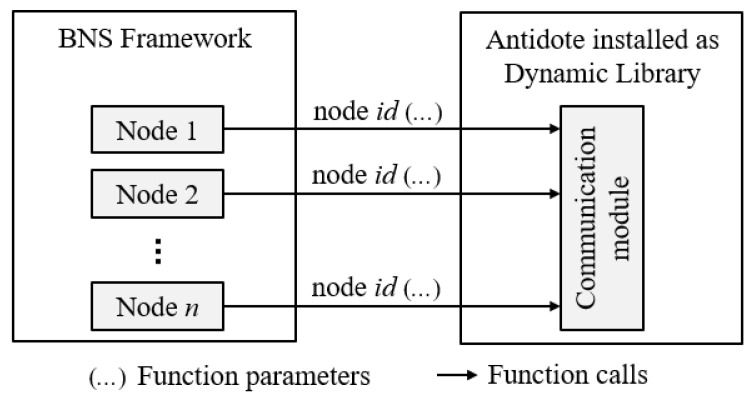
Communication module handling several nodes.

In the simulation environment, the Communication Module needs to deal with all nodes to control their transmissions. To ensure the proper operation of the Communication Module, the *node id* is required in each function call. This change in the Communication module is performed in our proposal. This way we know which node we are working with and apply the action for that node, without affecting other nodes. For example, if a node transits from disassociated state to associating state, without the proposed modification, this action would affect all nodes even those that should not change their state machines. Figure 7 illustrates the system modification. In this figure each arrow represents a function being called from the Communication Module. In every function in the Communication module, the *node id* new parameter is inserted. This way the Communication module always knows the corresponding node.

Other modules such as Agent, Manager and Encoders were modified to smooth operation of Antidote functions in the BNS framework. We can highlight the function created in the encoder module to convert absolute values (measurements) into scaled non-negative values as recommended by [48]. It is useful when we need to send absolute values such as −0.060 which would require at least 4 bytes in X73-PHD floating point format. When converted, the number −0.060 becomes 388, which requires just two bytes. Within the Manager, the conversion from scaled to absolute values is made [1].

## 4. Use Cases

In this section, three examples of use cases are presented, in which WBAN simulations are performed using the proposed BNS framework. In Section 4.1, a remote healthcare monitoring scenario is simulated using: a healthcare standard application, a WBAN MAC, a David wireless channel, body mobility and routing modules. In this example, we compare scenarios using the agent-initiated operation modes of ISO/IEEE 11073 standard and the Packet Delivery Rate (PDR) and latency are observed. Section 4.2 presents a comparison of the temperature behavior of the nodes of a WBAN using different routing protocols. This simulation uses a generic application, a WBAN MAC, a David wireless channel, body mobility and routing modules. Section 4.3 presents a simulation using a healthcare standard application, a WBAN MAC, a Goswami wireless channel and MoBAN proposed modules. In this use case, two channel access mechanisms defined in the WBAN standard for MAC layer are evaluated.

### 4.1. Remote Monitoring Using X73-Phd

**Figure 8:**
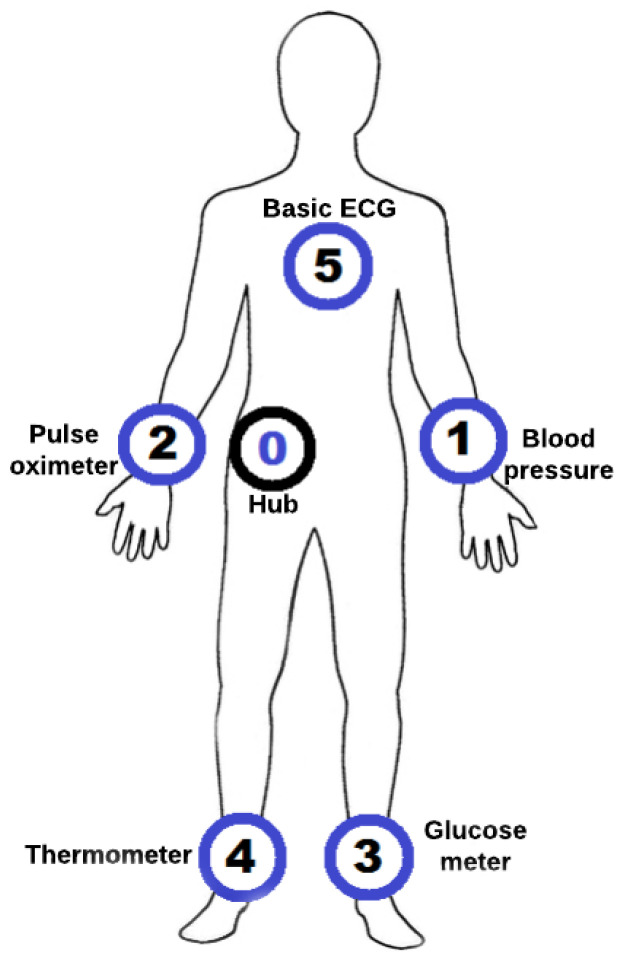
Network topology used in remote monitoring scenario.

A possible use case considered in this paper is a remote monitoring WBAN scenario, in which a patient’s vital signs are monitored using blood pressure monitor, thermometer, glucose meter, pulse oximeter and basic ECG. The network topology is formed by a coordinator node located in the waist and five PHD agents located in the body as shown in Figure 8. To model the channel behavior and consider body mobility, the loss map and the temporal variation model proposed in [23] are used. We used Direct Route Routing (DRR) recommended in the IEEE 802.15.6 standard. The PHY and MAC layers configurations used were those described in the WBAN standard and the MAC layer version used is the one provided in the BNS framework.

In this use case, we simulated three agent-initiated scenarios considering operating modes available in the application layer proposal. The three agent-initiated scenarios are: Unconfirmed Mode, Confirmed Mode, and a Retransmission Mode proposed in [1]. The node coordinator (hub) uses the *Manager* application. The blood pressure monitor transmits one measurement every 15 min. The oximeter sends a reading every 15 s. The glucose meter transmits one measurement every 5 min. The thermometer sends the temperature every 3 min. The ECG sensor considered is a device that receives signals of electrodes deployed in the body, and transmits these signals to the manager; so, it transmits 80 millivolt samples per 0.8 s. The total simulation time was 12 h.

**Figure 9:**
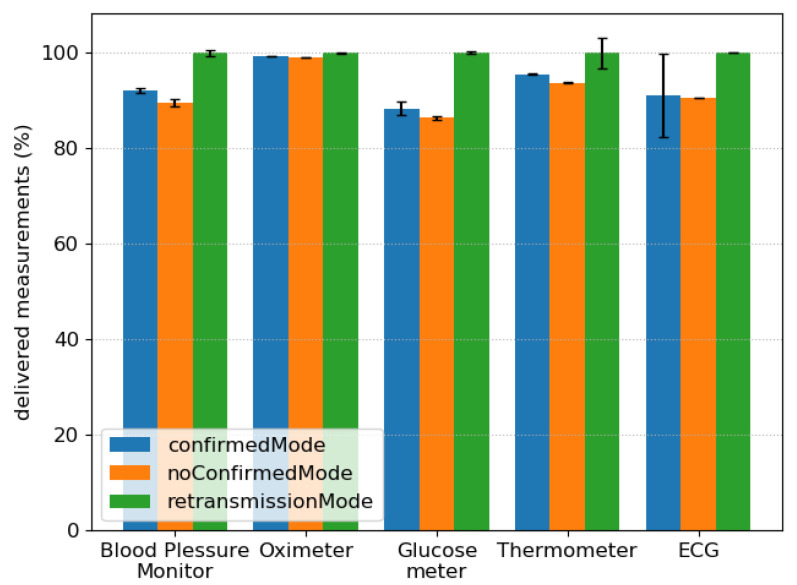
Measurement packets successfully delivered to the manager per node.

The results available in the application are the total control packets exchanged per node, the measurement packets received by the manager per node, the measurement packets sent by each node, and the total associations made per agent [1]. In this use case, we analyzed the total successful vital sign measurements delivered to the manager. Figure 9 depicts the results for these scenarios. As it can be seen in the figure, the proposed retransmission mode increases the number of packets delivered to all agents, delivering almost 100% of all messages. Additionally, it avoids the waiting time for a new association handshake, and by retransmitting the messages. For the oximeter, using the unconfirmed mode, almost all packets were delivered, even without guaranteeing reliability. However, the confirmed mode improves reliability, but it needs reassociations frequently. Results show that with the retransmission mode, the PDR in the WBAN network increases, offering better network performance for remote monitoring applications.

**Figure 10:**
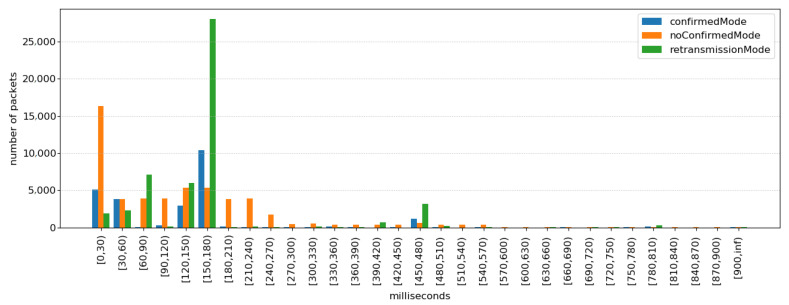
Distribution of packet’s latency in application layer.

An important network parameter to be considered when analyzing the performance of a WBAN is latency. Figure 10 depicts the number of packets with a given latency, for all measurements transmitted by agents in the simulations. The latency is calculated from the moment of packet creation, at the application layer of the agent, until the moment it is received at the application layer of the manager. The figure shows that most packets have an average latency of 180 ms in the retransmission mode and confirmed mode and 30 ms in no confirmed mode. Results show that the latency is maintained at acceptable values for remote monitoring applications.

This simulation scenario allows us to evaluate the performance regarding PDR and latency, using different modes of operation in the application layer, taking into consideration the effect of the wireless channel and the mobility of the human body. This comparative study is possible due to the resources made available in the proposed WBAN framework.

### 4.2. Analysis of Temperature Behavior

In WBAN, preventing nodes from overheating is a challenge that requires special attention. In [9], the Link-Quality Aware and Thermal-Aware On-Demand Routing (LATOR) protocol for routing in WBAN is proposed. LATOR is projected to avoid overheating and improve performance in sensing. For that, in [9] the authors propose a mechanism that chooses routes without heated nodes and when an intermediate node reaches the heated state, the choice of a new relay node is activated. That mechanism of overheating prevention is based on controlling the node’s activity, where a heated node does not carry out any transmission and enters a low consumption mode. On the other hand, Link Quality Information (LQI) is collected during the route discovery phase. Then, sensor nodes will use LQI-based metrics to forward DATA packets [9].

In this use case, a simulation is carried out to analyze the temperature behavior of the network nodes. The WBAN topology used is the same used in Section 4.1. The mobility of the body is considered, and the channel behavior used is the David channel model proposed in [23]. A generic sensor node was used in the application layer, with settings valid for medical sensors with low transmission rates. The PHY and MAC layer configurations used were those described in the WBAN standard and available in the BNS framework. Settings of the thermal parameters of the tissues of the human body are the same used in [9].

Similar to the work presented in [9], for comparison purposes in this use case, DRR, First Route Available Routing (FRAR), Link-Quality Aware On-Demand Routing (LAOR) and LATOR protocols were considered. These protocols are covered in [9], and in this paper we follow the same settings. To analyze the performance of the proposed overheating prevention method, a temperature increase limit of 0.0017 °C was defined for sensor nodes. The simulation time was ten thousand seconds. The metric observed was temperature increase.

**Figure 11:**
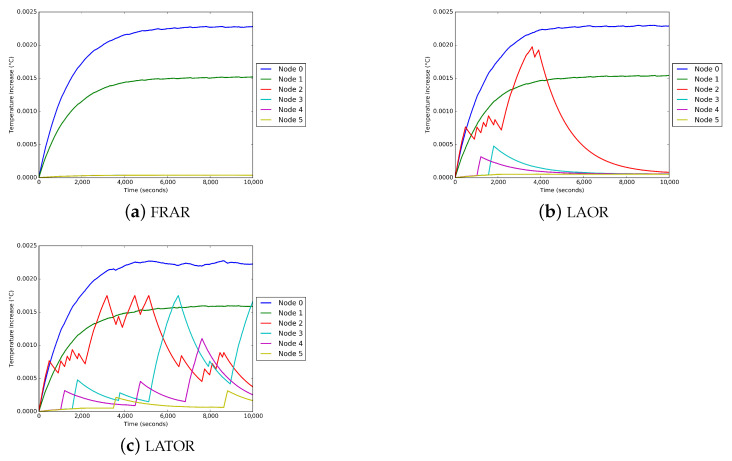
Temperature variation of the nodes. Node 1 acting as transmitter. (**a**) Using FRAR protocol; (**b**) using LAOR protocol; (**c**) using LATOR protocol.

For the purpose of this use case, the behavior of the temperature of the network nodes is analyzed when the node located in the left arm (node 1) is the transmitting node. Figure 11 shows the behavior of the temperature using different routing protocols. As it can be seen in Figure 11a, using the first available route, route 1-0 was used 100% of the time. Therefore, no intermediate node showed an increase in temperature. However, it can be seen in Figure 11b that for the LAOR protocol, node 2 experiences a greater increase in its temperature in the first 3800 s, because most of the time it acts as a relay node for node 1 on route 1-2-0. When the LATOR protocol is used, as shown in Figure 11c, the temperature of node 2 begins to increase until reaching the defined limit. This increase is due to the fact that node 2 is used more times as a relay, because route 1-2-0 has the best quality for forwarding the data transmitted by node 1. As a result, the temperature of node 2 begins to increase until it reaches the defined limit value. This value is reached for the first time at 3180 s.

At that moment, node 2 sends its heating notification to the transmitting node. Node 1, when it receives the notification, switches routes, and starts using the direct path. At that moment, node 2 starts to cool. Immediately thereafter, there are two more peaks at which node 2 again reaches the defined temperature limit. Between the first and the third peaks in the temperature of node 2, there were exchanges of routes, where the use of node 2 was resumed as a relay. In these cases, the change of route was not generated by heating, but by breaks in the link while using routes 1-0, 1-3-0 and 1-4-0. After the third temperature peak of node 2, route 1-3-0 begins to be used, until node 3 reaches the maximum allowed temperature. In this case, it is node 3 that activates a new route discovery to avoid overheating.

In this use case, we presented an example where the routing layer of a WBAN network uses the temperature module available in the proposed BNS framework to perform temperature control and prevent overheating. This temperature module can be used by other modules in other proposals. This simulation scenario shows the temperature variation of the nodes due to the activity of the node, the link breaks due to body mobility and the influence of the wireless channel on the quality of the links. All these features are covered by the application, routing, mobility and wireless channel modules, available in the proposed WBAN framework. The results show the correct functioning of the proposed temperature module and allows considering temperature as a parameter in the performance analysis of WBAN solutions.

### 4.3. Analysis of Channel Access Mechanisms

**Figure 12:**
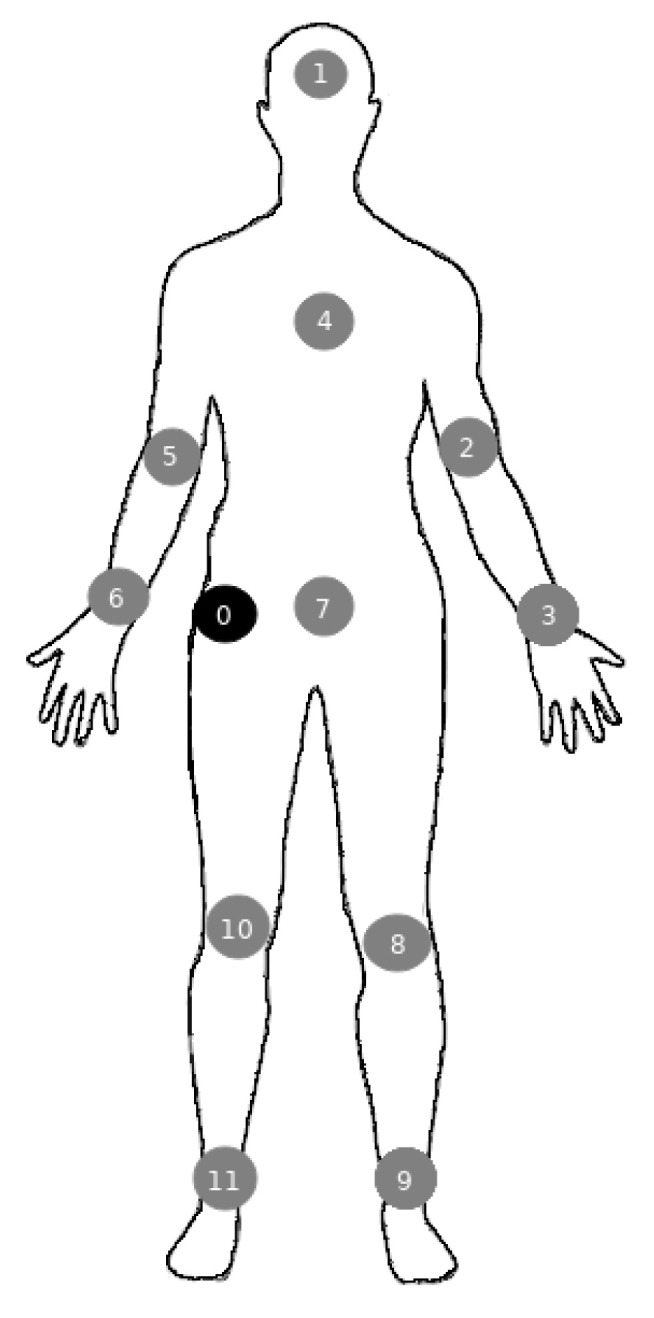
WBAN topology with 12 nodes.

In the MAC layer of the WBAN standard, the use of the TDMA and CSMA/CA mechanisms are accepted, for data transmission at the scheduled intervals and random access, respectively. In this use case, a comparison of the performance of a WBAN using Time Division Multiple Access (TDMA) and CSMA/CA techniques is presented. For this comparison, a WBAN scenario with 12 health devices distributed in the human body was simulated. The positions of the nodes are represented in Figure 12 and in Table 3 the health devices and their data transmission rates are described.

The application QoS requirements used in this scenario are of intelligent health applications [49,50] and the dynamics of the application’s data flow is based on the unconfirmed mode of the ISO/IEEE 11073 standard available in the Healthcare Application Layer of the proposed BNS framework.

The nodes were in walking motion for 10 min of simulation. To simulate the mobility of the human body, the MoBAN proposed module was used, and the behavior of the channel was modeled using the Goswami wireless channel. In the PHY layer, the radios have a transmission power of −15 dBm and the transmission rate used is the maximum allowed for a radio compatible with the standard NB-PHY, 1024 kb/s. In the MAC layer, the slot allocation time is used as a minimum value for IEEE 802.15.6 NB-PHY, which is 1 ms. Each node when joining the network requests 4 slots for transmission. As for the size of the superframe, 32 allocation slots were made available. Therefore, the time between beacons, i.e., the expected duration of a superframe is 92 ms. The metrics observed are the PDR of the application and the energy used to transmit a bit from the application (μJ/bit). The results shown have a 95% confidence interval for ten simulation rounds.

**Figure 13:**
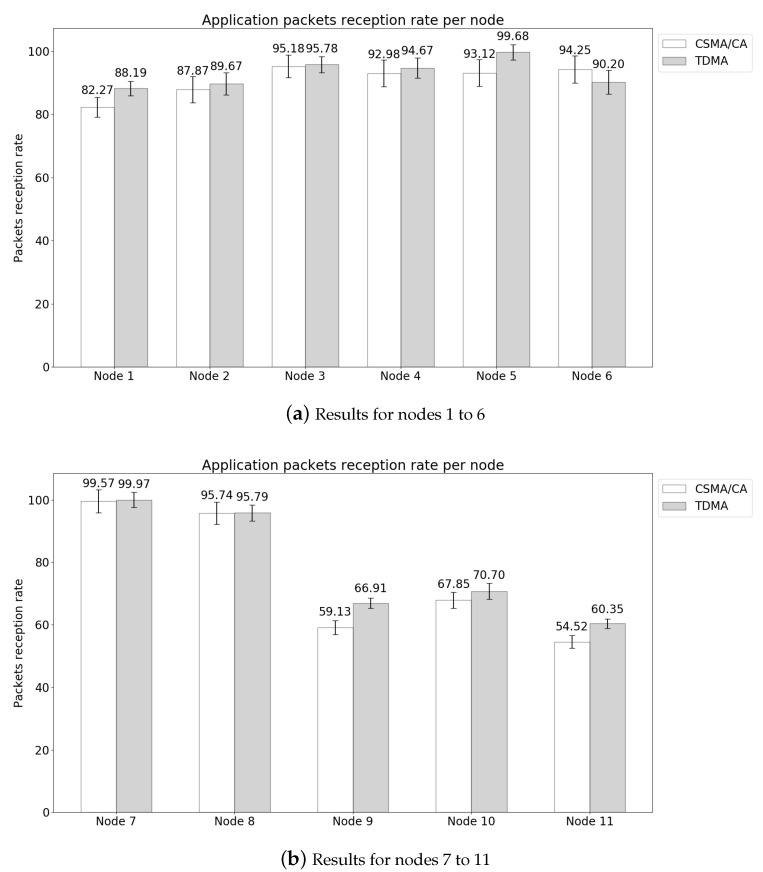
Application PDR for the 12 nodes topology.

The results of the application PDR per node are represented in Figure 13. Figure 13a contains the results for the nodes positioned on the head, chest and upper limbs, whereas the results of Figure 13b are of the nodes in the waist and lower limbs. The PDR of lower limbs are inferior to those of the upper limbs, given the distance and the pendulum movement of the legs during walking, which increases the path loss when there is no line-of-sight to the hub. Another interesting fact is the synchronization of movements between the right arm/left leg and left arm/right leg, natural during the walking motion. This synchronized movement plays an important role in the wireless channel response, with nodes in the left leg having a better reception rate than the nodes in the right one, given the interference of nodes near the hub. Additionally, the TDMA scheduling and the pendulum movement cycle can influence the PDR, and proper transmission scheduling can take advantage of that periodic behavior [4]. The smaller interference of nodes in the right arm and a better transmission scheduling can be observed in Node 8, even though it is placed in an inferior limb, it has a high PDR. In this scenario we can highlight that the average PDR using TDMA is superior to using CSMA/CA, and considering the error margin, they have similar results. Another interesting fact is that for node 6, which is closer to the hub, CSMA/CA results are superior, therefore under good channel conditions CSMA/CA can improve the network throughput. The overall network PDR results were 86.54±2.58% for TDMA, and 83.86±3.43 for CSMA/CA.

**Figure 14:**
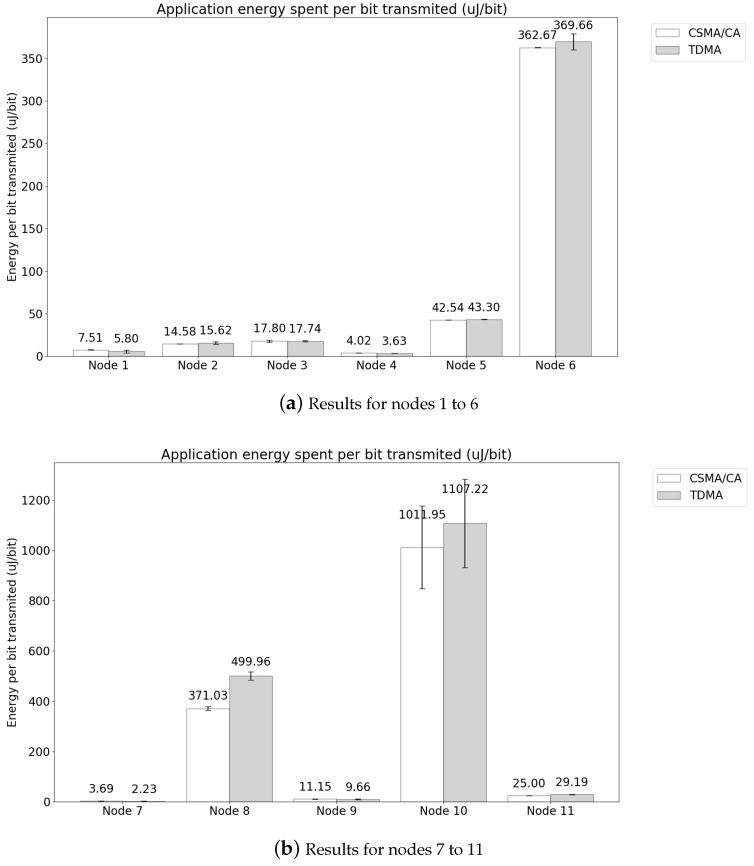
Energy spent per transmitted application bit in 12 nodes topology.

The results for energy spent per transmitted application bit are represented in Figure 14. As in the results of the application PDR, the nodes were divided between nodes positioned on the head, chest and upper limbs in Figure 14a, and nodes in the waist and lower limbs in Figure 14b. The low PDR of lower limbs incurs in retransmissions and increases the energy spent to transmit a bit. In addition, smaller packets also increase the energy spent to transmit an application bit. Those influences can be observed in Node 10, with a low PDR and low transmission rate, but a high energy spent to transmit an application bit. The results are consistent with the PDR analysis, and the results of both MAC methods are similar. It is important to highlight the application influence on energy consumption. The result of energy spent per transmitted application bit for the network was 180.17±19.14 μJ/bit for TDMA and 170.48±21.75μJ/bit for CSMA/CA.

This simulation scenario shows that energy consumption and network PDR heavily rely on node deployment, mobility, wireless channel and the application itself. All those features are covered by the MoBAN, Goswami channel, and IEEE 11073 modules, respectively. Therefore, to design a communication protocol for WBANs, those characteristics particular to the WBAN scenario should be considered.

## 5. Conclusions

Performing evaluations in environments as close as possible to real scenarios is necessary for WBAN. To provide a realistic WBAN simulation system, this work proposes the BNS framework for developing WBAN realistic simulations. In contrast to the existing WBAN simulation systems, additional modules were implemented to provide greater flexibility in the construction of WBAN scenarios. The BNS framework includes a wireless channel model considering radio-propagation on human body; an updated WBAN MAC layer with functionalities of the IEEE 802.15.6 standard; a mobility model for simulating intra-WBAN communication; a temperature module to model node temperature variation; and a Healthcare Application Layer according to the ISO/IEEE 11073 standard.

We presented three WBAN simulations using the BNS framework: a remote healthcare monitoring scenario using the application layer based on the ISO/IEEE 11073 standard, a generic scenario to analyze the temperature behavior of the nodes of a WBAN using different routing protocols and a comparison of the performance of a WBAN using TDMA and CSMA/CA techniques. These simulations are examples that show the use of the proposed BNS framework and show the flexibility to build different realistic scenarios, concluding that our framework is a valid alternative for the evaluation of WBAN through simulation.

The BNS framework is focused on communication between sensors/actuators nodes and the sink node (intra-WBAN communication). Technologies such as 3G/4G/5G/6G are alternatives to use in Inter-WBAN and Beyond-WBAN communications. Thus, the sink node with the corresponding radio interface acts as an aggregator of the information collected by the sensor nodes, performing its role as a gateway. To perform simulations considering the different levels of the WBAN architecture defined in the standard (including Inter-WBAN and Beyond-WBAN), it is necessary to continue improving the proposed framework, which would be an interesting future work. As future work, we also intend to increase the support for other types of personal health devices in the BNS framework application layer; support other body mobility patterns; additional WBAN channel models and provide the IEEE 802.15.6 security support. With that, the framework could support a greater number of configurations and increase the diversity of WBAN scenarios. Moreover, we intend to develop a real testbed with real personal health devices, which would allow the performance of realistic emulations, in addition to creating new models to be implemented in the BNS framework.

## Figures and Tables

**Table 1:** Comparison of WBAN simulation platforms.

Simulation Platform	Body Mobility	Wireless Channel	Temperature Model	IEEE 802.15.6 MAC Layer	IEEE 802.15.6 PHY Layer	Health Devices (X73-PHD)
OMNET++ [25]	Yes	No	No	No	No	No
Castalia [12,32,33,34,35]	No	Yes	No	Yes (partially)	Yes	No
MiXiM [13]	Yes	No	No	No	No	No
NS-2 [14,15,39,40]	No	No	No	No	No	No
NS-3 [16,42,43]	No	No	No	Yes	No	No
Matlab [17,18,19,20,21]	No	Yes	Yes	No	No	No
BNS Framework	Yes	Yes	Yes	Yes	Yes	Yes

**Table 2:** BNS Healthcare Application Layer Configurable Simulation Parameters.

Parameter Name	Value
ApplicationName	“Manager”, “Agent”
hubNode	double value
application_type	“pulseoximeter”, “thermometer”, “bloodpressure”, “basicECG”, “glucometer”
measurements_per_second	double value
confirmed_event	true, false
retransmitPacket	true, false
maxNumOfRetransmission	integer value
timeOutToRetransmitPacket	double value
managerInitiated	true, false
managerInitiateMode	“singleMode”, “timePeriodMode”, “noTimePeriodMode”
managerInitiatedTime	double value. Used in “timePeriodMode”
numberOfReceived, MeasurementsToSendStop	integer value. Used in “noTimePeriodMode”

**Table 3:** WBAN sensors nodes, their positions and transmission rates.

Node	Position	Node Type	Transmission Rate
0	Right waist	Hub	-
1	Head	EEG	86.5 Kb/s
2	Left arm	Blood pressure	1.2 Kb/s
3	Left hand	Glucose Monitor	1 Kb/s
4	Chest	ECG	192 Kb/s
5	Right arm	Blood flow	480 b/s
6	Right hand	Heart rate meter	48 b/s
7	Center of waist	Medication injector	16 Kb/s
8	Left leg	blood pH	48 b/s
9	Left foot	Motion sensor	35 Kb/s
10	Right leg	Temperature sensor	2.4 b/s
11	Right foot	GPS	96 b/s

## Abbreviations

The following abbreviations are used in this manuscript:

ACK	Acknowledgment
BHTE	Bioheat Transfer Equation
BMI	Body Mass Index
BNS	Body Network Simulator
CSMA/CA	Carrier Sense Multiple Access with Collision Avoidance
DIM	Domain Information Model
DMQoS	Data-Centric Multiobjective QoS-Aware Routing
DRR	Direct Route Routing
ECG	Electrocardiogram
FRAR	First Route Available Routing
LAOR	Link-Quality Aware On-Demand Routing
LATOR	Link-Quality Aware and Thermal-Aware On-Demand Routing
LOS	Line-of-Sight
LQI	Link-Quality Information
MAC	Medium Access Control
MoBAN	Mobility Model for Wireless Body Area Networks
NB-PHY	Narrow Band Physical Layer
NLOS	Non-Line-of-Sight
NS-2	Network Simulator 2
NS-3	Network Simulator 3
OEP	Optimized Exchange Protocol
OMNET++	Objective Modular Network Testbed in C++
PDF	Probability Density Function
PDR	Packet Delivery Rate
PHD	Personal Health Device
PHY	Physical Layer
PPS	Packets Per Second
QoS	Quality of Service
RERR	Route Error
RREQ	Route Request
RSSI	Received Signal Strength Indicator
RT	Routing Table
Rx	Receiver
SAR	Specific Absorption Rate
SINR	Signal-to-Interference-plus-Noise Ratio
TDMA	Time Division Multiple Access
Tx	Transmitter
UWB	Ultra-wideband
UWB-PHY	Ultra-wideband Physical Layer
X73-PHD	ISO/IEEE 11073 Personal Health Devices
WBAN	Wireless Body Area Networks
WSN	Wireless Sensor Network
